# Prevalence of Psychopathological Symptoms and Their Determinants in Four Healthcare Workers’ Categories during the Second Year of COVID-19 Pandemic

**DOI:** 10.3390/ijerph192013712

**Published:** 2022-10-21

**Authors:** Alessandra Gorini, Mattia Giuliani, Elena Fiabane, Alice Bonomi, Paola Gabanelli, Antonia Pierobon, Pasquale Moretta, Giovanna Pagliarulo, Simona Spaccavento, Gaetano Vaudo, Matteo Pirro, Massimo R. Mannarino, Laura Milani, Maria Paola Caruso, Paola Baiardi, Laura Adelaide Dalla Vecchia, Maria Teresa La Rovere, Caterina Pistarini, Damiano Baldassarre

**Affiliations:** 1 Department of Clinical Sciences and Community Health, University of Milan, 20122 Milan, Italy; 2 Istituti Clinici Scientifici Maugeri IRCCS, Milano-Camaldoli, 64, 20138 Milan, Italy; 3 Centro Cardiologico Monzino, IRCCS, 20138 Milan, Italy; 4 Department of Physical and Rehabilitation Medicine, Istituti Clinici Scientifici Maugeri IRCCS, 16167 Genoa, Italy; 5 Istituti Clinici Scientifici Maugeri IRCCS, Psychology Unit of Pavia Institute, 27100 Pavia, Italy; 6 Istituti Clinici Scientifici Maugeri IRCCS, Psychology Unit of Montescano Institute, 27040 Montescano, Italy; 7 Istituti Clinici Scientifici Maugeri IRCCS, Neurological Rehabilitation Unit of Teleselezioni Terme Institute, 82037 Telese Terme, Italy; 8 Istituti Clinici Scientifici Maugeri IRCCS, Psychology Unit of Bari Institute, 70124 Bari, Italy; 9 Department of Medicine and Surgery, University of Perugia, 06123 Perugia, Italy; 10 Unit of Internal Medicine, “Santa Maria” Terni University Hospital, 05100 Terni, Italy; 11 Unit of Internal Medicine, Department of Medicine, University of Perugia, 06132 Perugia, Italy; 12 ASST Crema, 26013 Crema, Italy; 13 SITRA, Policlinico San Donato, 20097 Milan, Italy; 14 Istituti Clinici Scientifici Maugeri IRCCS, Direzione Scientifica Centrale of Pavia Institute, 27100 Pavia, Italy; 15 Istituti Clinici Scientifici Maugeri IRCCS, Department of Cardiology of Montescano Institute, 27040 Montescano, Italy; 16 Istituti Clinici Scientifici Maugeri IRCCS, Department of Neurorehabilitation of Pavia Institute, 27100 Pavia, Italy; 17 Department of Medical Biotechnology and Translational Medicine, University of Milan, 20122 Milan, Italy

**Keywords:** impact of COVID-19, healthcare workers, psychopathological symptoms, post-traumatic stress disorder, burnout

## Abstract

Highly stressful situations, such as the current COVID-19 pandemic, induce constant changes in the mental state of people who experience them. In the present study, we analyzed the prevalence of some psychological symptoms and their determinants in four different categories of healthcare workers during the second year of the pandemic. A total of 265 physicians, 176 nurses, 184 other healthcare professionals, and 48 administrative employees, working in different Italian healthcare contexts, answered a questionnaire including variables about their mental status and experience with the pandemic. The mean scores for anxiety and depressive symptoms measured more than one year after the onset of the pandemic did not reach the pathological threshold. In contrast, post-traumatic and burnout symptoms tended toward the critical threshold, especially in physicians. The main determinant of psychological distress was perceived stress, followed by job satisfaction, the impact of COVID-19 on daily work, and a lack of recreational activities. These results increase the knowledge of which determinants of mental distress would be important to act on when particularly stressful conditions exist in the workplace that persist over time. If well-implemented, specific interventions focused on these determinants could lead to an improvement in employee well-being and in the quality of care provided.

## 1. Introduction

Two years have passed since researchers in China identified a new virus, called SARS-CoV-2 causing COronaVIrus Disease 2019 (COVID-19), that initially infected dozens of people in Asia. Since then, the virus has infected more than 80 million people worldwide, and more than 1.7 million people have died. In Italy alone, the number of cases has already exceeded 5.4 million, with more than 136,000 deaths. Healthcare workers have undoubtedly played a pivotal role in managing the pandemic, but their professional and personal lives have been significantly disrupted since the beginning. Their daily workload has significantly increased, and many have been reassigned to areas outside their clinical expertise, with frequent changes in roles and responsibilities [1,2]. As if that were not enough, healthcare providers have been forced, much more than others, to forgo social contacts and recreational activities to protect their families and themselves (and consequently, their patients) from contagion. Such prolonged physical and mental stressors have exposed healthcare workers to a significantly increased risk of developing psychological disorders such as anxiety, depression, stress, and insomnia, as is well-documented by several studies and meta-analyses published in 2021 [3,4,5]. Anxiety and depression, as well as somatization and insomnia, were higher in physicians, nurses, and nonmedical staff than in professionals in other areas [6,7] and in the general population [8,9]. Data published in 2020 also show that, among healthcare workers, women were more predisposed to develop stress [10], and nurses were at increased risk of depression and anxiety [10,11], especially for women with poor self-efficacy, resilience, and social support and with pre-existing physical symptoms [12]. Pooled prevalence estimates of depression and anxiety were highest in studies conducted in the Middle East (34.6%; 28.9%) [13].

The symptoms and psychological disorders described above relate primarily to the first year of the pandemic’s spread. Unfortunately, the spread of the virus continues, and the pandemic’s significant impact on healthcare systems around the world persists. Persistent stress, work overload, and emotional burden are known to cause severe mental disorders, such as post-traumatic stress disorder and burnout.

With these premises, the present research aimed to evaluate the long-term psychological impact of the pandemic’s persistence in a sample of Italian healthcare workers during the first half of the second year of the pandemic. Specifically, we focused on symptoms related to depression and anxiety that may arise as a consequence of exposure to dramatic events, as well as on post-traumatic stress disorder and burnout, that typically emerge in stressful conditions persisting over time, and analyzed their main determinants and if they differed among various categories of healthcare workers. Our main hypothesis is that post-traumatic stress and burnout symptoms are present in healthcare professionals after more than a year of pandemic spread due to a prolonged exposure to acute and chronic stress.

## 2. Materials and Methods

### 2.1. Participants

The data were collected using an online survey distributed through Qualtrics software (Provo, UT, USA) during the third COVID-19 pandemic wave in Italy (i.e., from 1 March to 16 July 2021). An anonymized, individual, and unique code to complete the survey was provided for those who agreed to participate in the study. Employing convenient sampling, healthcare workers practicing in several Italian hospitals and healthcare institutions were invited to carry out the survey. To promote the survey, participants were encouraged to pass the link to other colleagues.

**Figure 1:**
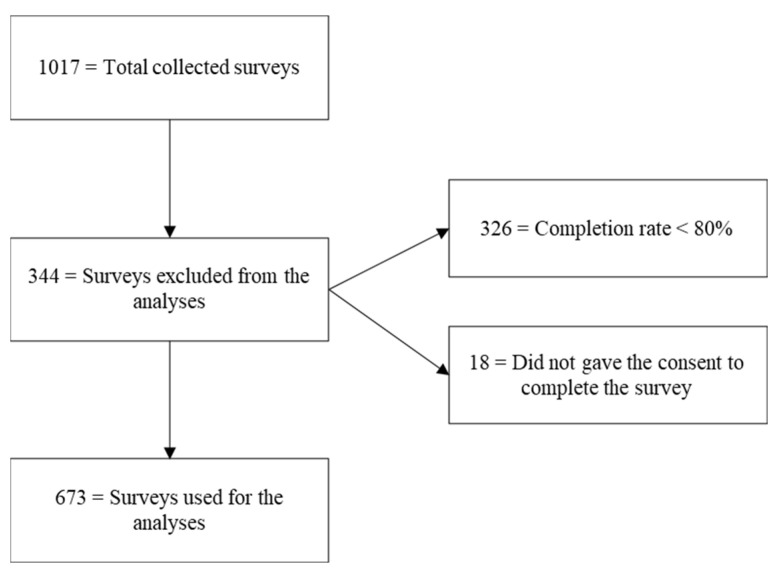
Flow chart.

A total of 1017 surveys were collected. Of these, 673 (62.2%) were deemed suitable for the analyses. Of the remaining 344 (37.8%), 326 (94.8%) were excluded because participants completed less than 80% of the survey, and 18 (5.2%) because they declined the consent to participate (see Figure 1). More than 1/3 of those who completed less than 80% of the survey (*n* = 140) just electronically signed the informed consent without answering any other question, while 2/3 of them (*n* = 225) answered fewer than the first 6 questions (i.e., gender, age, marital status, health status). For these reasons, the incomplete questionnaires could not be included in the analyses.

Once participants entered the questionnaire, they were forced to answer all the questions consecutively, and allowed to “stop and save” the survey and complete it in more than one session. Once the survey was completed, the link expired, preventing participants from responding more than once. Subjects also had the option to quit the survey at any time.

Inclusion criteria were: (a) age ≥ 18 years, (b) being a native Italian speaker, and (c) being a healthcare worker or working in a healthcare institution.

The present study was approved by the local Scientific Ethics Committee (approval number 2411, 26 March 2020 and subsequent amendments), and all participants provided informed consent to participate. The survey was anonymous, and confidentiality of information was ensured.

### 2.2. The Survey

The survey included the following domains: (a) sociodemographic information (i.e., age, sex, and marital status); (b) physical health status (i.e., presence/absence of organic disease and type of disease); (c) work-related information (i.e., employment, institution name, average working hours in the last four weeks, and job seniority); (d) perceived risk and fear of COVID-19-related infection before and after the vaccination; (e) the perceived impact of COVID-19 on workplace and individual working activities; (f) psychological status (e.g., symptoms of anxiety, depression, post-traumatic disorder, and burnout). See File S1 for the complete version of the survey.

#### 2.2.1. Assessment of Anxiety and Depression Symptoms

Symptoms of anxiety and depression were evaluated using the 4-item Patient Health Questionnaire (PHQ-4), which is a validated ultrabrief tool with a good internal reliability, construct validity, and factorial validity, to identify potential cases of depression and anxiety [14]. The PHQ-4 consists of the first 2 items of the 7-item Generalized Anxiety Disorder (GAD-7) [15] and the first 2 items of the 9-item Patient Health Questionnaire (PHQ-9) [16]. Responses are provided on a Likert scale ranging from 0 (not at all) to 3 (nearly every day). A total score ≥ 6 for the PHQ-4, or ≥3 for the two subscales, indicates the presence of mild symptoms, while a total score ≥ 9 for the PHQ-4, or ≥5 for the two subscales, indicates the presence of severe symptoms [14]. Due to its excellent operating characteristics, the PHQ-4 is considered a valid substitute for the two original scales (GAD-7 and PHQ-9) to assess both anxiety and depression in busy clinical and nonclinical settings and conditions, such as the COVID-19 emergency, in which healthcare providers had a very short time to complete questionnaires.

#### 2.2.2. Assessment of Post-Traumatic Distress Symptoms

Post-traumatic distress symptoms caused by the COVID-19 pandemic were assessed using the Impact of Event Scale-Revised (IES-R), a validated self-report questionnaire composed of 22 items [17]. The IES-R includes three subscales measuring the following dimensions: intrusion, avoidance, and hyperarousal. Participants were asked to rate their level of distress using a 5-point Likert scale ranging from 0 (not at all) to 4 (often) referring to the previous seven days. The total score ranges from 0 to 88, with the following cutoff for score interpretation: 0–23 indicates the absence of relevant symptoms; 24–32 indicates the presence of mild symptoms; 33–36 indicates the presence of moderate symptoms; and 37–88 indicates the presence of severe post-traumatic distress symptoms.

#### 2.2.3. Assessment of Burnout Symptoms

Burnout symptoms were measured using the Maslach Burnout Inventory—General Survey (MBI-GS), which is a validated 16-items self-report questionnaire [18]. The MBI-GS provides a total score, as well as the following three subscales: “Exhaustion”, “Cynicism”, and “Professional Efficacy”. All the items are scored on a seven-point Likert scale, ranging from 0 (“never”) to 6 (“every day”). The degree of burnout is high when the scores for exhaustion and cynicism are high and the score in professional efficacy is low.

### 2.3. Statistical Analysis

Continuous variables are presented as mean ± standard deviation. Comparisons were performed using ANOVAs. Variables with a skewed distribution were compared with the Wilcoxon rank-sum test, and they are presented as median and interquartile ranges. Categorical data were compared using χ^2^ test or Fisher‘s exact test, as appropriate, and they are reported as frequency and percentage.

Independent predictors of psychological outcomes were identified by multiple linear regression analyses with stepwise selection of the variables (the stepwise regression results are shown in the supplementary materials). The consistency and reliability of the identified subset of predictors were tested by a cross-validation iteration procedure. At each step, the dataset was randomly split into two halves. The independent predictors were selected in the first half (training set), and the resulting model was tested for significance in the second half (testing set). The procedure was repeated 200 times, each one with a different random split. A predictor was deemed to be reliable if selected and confirmed in at least 70% of the time.

The data are presented as Beta (β) ± standard error.

*p*-Values < 0.05 were considered as significant and all tests were two-sided. Analyses were performed using SAS statistical package V. 9.13 (SAS Institute, Inc., Cary, NC, USA).

## 3. Results

### 3.1. Descriptive Statistics

The respondents included 265 physicians, 176 nurses, 184 other healthcare professionals (i.e., psychologists, physiotherapists, dieticians, and speech therapists), and 48 administrative employees. The largest proportion of women was found in the group of physicians (83.4%), while the administrative staff group was the one with the highest mean age (mean = 47.28 ± 10.71 years). Almost half of the workers in each group were married (physicians: 49.6%; nurses: 49.4%; other healthcare professionals: 45.9%; administrative staff: 54.2%). Regarding psychological variables, symptoms of depression, anxiety, and burnout did not differ among the four groups, while post-traumatic stress was higher in the physicians’ group (see Table S1 for the clinical cutoff scores and Tables S2–S5 in the Supplementary Materials for the stepwise logistic regression results). 

All collected variables are shown in Table 1.

### 3.2. Cross-Validation Procedure

#### 3.2.1. Symptoms of Depression (PHQ-4)

Among possible predictors, only perceived stress and perceived job satisfaction (both referred to the last two weeks) survived the cross-validation procedure. Specifically, for all the groups, a higher perceived stress predicted higher symptoms of depression (physicians: β = 0.024 ± 0.003, *p*-value < 0.0001; nurses: β = 0.013 ± 0.004, *p*-value < 0.0001; other healthcare professionals: β = 0.018, SE = 0.003, *p*-value < 0.0001; administrative staff: β = 0.025 ± 0.004, *p*-value < 0.0001). Conversely, a higher perceived job satisfaction predicted lower symptoms of depression only in the physicians and nurses’ groups (physicians: β = −0.015 ± 0.003, *p*-value < 0.0001; nurses: β = −0.016 ± 0.004, *p*-value < 0.0001). The cross-validation results are shown in Table 2.

#### 3.2.2. Symptoms of Anxiety (PHQ-4)

Among possible predictors, only perceived stress and perceived job satisfaction (both referring to the last two weeks) survived the cross-validation procedure. Specifically, for all the groups, a higher perceived stress predicted higher symptoms of anxiety (physicians: β = 0.028 ± 0.003, *p*-value < 0.0001; nurses: β = 0.022 ± 0.003, *p*-value < 0.0001; other healthcare professionals: β = 0.023 ± 0.003, *p*-value < 0.0001; administrative staff: β = 0.035 ± 0.005, *p*-value < 0.0001). Conversely, a higher perceived job satisfaction predicted lower symptoms of anxiety only in the physicians’ group (β = −0.015 ± 0.003, *p*-value < 0.0001). The cross-validation results are shown in Table 3.

#### 3.2.3. Post-Traumatic Symptoms (IES-R)

Among possible predictors, only perceived stress during the last two weeks, having started psychological support since the pandemic’s beginning, and lack of recreational activities after work survived the cross-validation procedure. In particular, for physicians, nurses, and other healthcare professionals, higher perceived stress predicted higher post-traumatic stress symptoms (physicians: β = 0.183 ± 0.040, *p*-value < 0.0001; nurses: β = 0.160 ± 0.034, *p*-value < 0.0001; other healthcare professionals: β = 0.147 ± 0.039, *p*-value < 0.0001). Having started psychological support since the pandemic’s beginning predicted higher post-traumatic stress symptoms in the nurses’ group (β = 7.315 ± 1.468, *p*-value < 0.0001), while the lack of recreational activities after work due to COVID-19 predicted higher post-traumatic symptoms only in the group of administrative workers (β = 0.301 ± 0.0460, *p*-value 0.0021). The cross-validation results are shown in Table 4.

#### 3.2.4. Burnout Symptoms

Among the possible predictors, only perceived stress during the last two weeks and the impact of COVID-19 on daily work survived the cross-validation procedure. Interestingly, no variables survived the cross-validation in both other healthcare professionals and the administrative workers. For both the physicians and the nurses’ groups, a higher perceived stress predicted higher burnout symptoms (physicians: β = 0.146 ± 0.028, *p*-value < 0.0001; nurses: β = 0.140 ± 0.030, *p*-value < 0.0001). Furthermore, perceiving that COVID-19 was still impacting daily work predicted higher burnout symptoms in the physicians’ group only (β = 2.635 ± 0.666, *p*-value < 0.0001). The cross-validation results are shown in Table 5.

## 4. Discussion

Data collected around the world during the first year of the COVID-19 pandemic have shown an increased prevalence of anxiety, depression, and sleep disturbances in healthcare workers [19,20,21]. In addition, a large body of literature suggests that prolonged and chronic stress negatively affects both psychological and physical health, inducing severe mental health conditions, such as post-traumatic stress disorder and burnout [22,23,24,25]. In the presence of a lasting emergency, such as that imposed by COVID-19, this evidence underscores the importance of monitoring the long-term psychological consequences on healthcare workers over time as well as identifying the determinants of these alterations with multiple studies conducted at different stages of the pandemic.

In this study, we analyzed the prevalence of anxiety, depression, burnout, and post-traumatic symptoms in four categories of healthcare providers involved in the COVID-19 emergency for more than a year. Partially in accordance with our main hypothesis that predicted an increase in post-traumatic and burnout symptoms in all the healthcare providers after more than a year of pandemic, we found that physicians showed mild post-traumatic symptoms which were significantly higher than in the other three groups. Anxiety, depression, and burnout symptoms did not differ among the four groups.

During the first wave of the pandemic, great resonance was given to the fact that nurses suffered from psychopathological symptoms more than other healthcare categories, mainly due to the initial lack of personal protective equipment (PPE), the increased workload, and their strict and prolonged physical and moral closeness to patients [26,27]. Two recent meta-analyses have confirmed these data [10,11]. However, it is plausible that after more than a year from the start of the emergency, a sort of “habituation” effect has occurred which has led the different categories of operators to experience milder anxiety and depressive symptoms.

In terms of the differences found in post-traumatic symptoms, physicians seem to be the most affected. The significant difference observed when comparing physicians and other healthcare professionals, which, to our knowledge, has not been investigated in previous studies performed during the COVID-19 pandemic, might be explained by considering the main independent risk factors for post-traumatic disorders in healthcare, which are (i) working on the front line; (ii) being under occupational pressure; (iii) receiving a low level of support from the hospital administration; and (iv) having greater responsibility for frontline clinical care [28,29]. Especially during exceptional events such as the current pandemic, such conditions typically affect frontline healthcare workers, doctors in particular, who, more than the others, have to take on the decision-making responsibility of patients’ lives. Moreover, it should be considered that the prevalence of post-traumatic symptoms may continue to increase even after the acute phase of the pandemic passes, suggesting the urgent need to develop potentially beneficial preventative programs for the individuals [30].

Our data show that perceived stress experienced during the last two weeks was the main predictor of (a) depressive and anxious symptoms in all the categories considered, (b) post-traumatic symptoms in all workers except the administrative staff, and (c) burnout symptoms in physicians and nurses. A strong association between stress and both individual psychophysical and occupational health has been observed in other studies carried out during the pandemic emergency [27,31,32,33]. A relevant role in the prediction of depressive (in physicians and nurses) and anxiety (in physicians) symptoms may also be attributed to job satisfaction, which is a well-recognized factor influencing the workers’ health [34,35,36].

The impact of COVID-19 on daily work is also a significant predictor of burnout in physicians. That this effect is specific to physicians is not surprising. More than others, this category of healthcare professionals has been asked to act outside of their own usual role to fulfill pandemic-induced needs [37].

Finally, the lack of recreational activities emerged as a potential predictor of post-traumatic symptoms in the administrative staff. It can be assumed that, before the spread of the pandemic, workers belonging to this category had more time than others to devote to leisure activities and having to give them up was a significant factor in their malaise. Seeking creative expression and mental stimulation, keeping fit, and maintaining social connections have been proven to predict higher well-being [38]. On the contrary, being limited or unable to participate in their activities, especially during a highly stressful condition such as a pandemic, may become a significant determinant for the development of post-traumatic symptoms. Moreover, administrative staff do not have direct contact with the patients, and therefore their possible mental health problems are not related to those linked to frontline roles, but rather to the general limitations caused by the pandemic.

Being one of the first studies conducted more than a year after the beginning of the COVID-19 pandemic, the present study has the merit of having analyzed the long-term consequences of a highly stressful condition that has had (and is still having) heavily disrupted healthcare facilities. Nevertheless, it is limited by its cross-sectional nature, the drop-out rate, and the fact that we were not able to test the entire population of healthcare providers working in the selected hospitals and healthcare institutions. Regarding the cross-sectional nature, it imposes caution in the generalization of the results, derived from a nonrandomized sample and obtained through screening measures. In addition, the cross-sectional nature of the study significantly limits causal explanations.

Regarding the drop-out rate, since more than 1/3 of the excluded responders just electronically signed the informed consent without answering any of the other questions, while 2/3 of the excluded responders answered fewer than the first six questions, we were not able to statistically describe the excluded sample. Nevertheless, the response rate obtained in the present study is consistent with findings presented by Hoerger [39], who found that 10% of participants completing one of six online research surveys dropped out of the study almost immediately, with a linear rate of 2% dropout per 100 survey items presented. Moreover, considering responders who answered more than 80% of questions, we had a completion rate of 66%, which is higher than the mean survey completion rate that is generally between 20–50%.

Moreover, the fact that our sample was limited to volunteers increases the risk of the presence of some biases in the results that may affect and distort inference and make the generalizability of the results questionable. Although effective control over selection bias in surveys, including volunteers, is virtually impossible, its impact on the survey results is impossible to predict. To limit such biases, future surveys could include, whenever possible, a small component of a random sample to assess the presence and potential effects of selection bias.

Finally, acute and chronic stressors unrelated to COVID-19 were not evaluated and should be considered in future studies.

## 5. Conclusions

In conclusion, our data, collected after a long period of exposure to a particularly traumatic situation such as an enduring pandemic, show that, in the long term, only post-traumatic and burnout symptoms tend to reach the critical threshold, and this is especially true in physicians. In addition, our data also document that the main determinants of psychological distress are perceived stress, followed by job satisfaction, the impact of COVID-19 on daily work, and lack of recreational activities.

We believe that these findings, as well as broader considerations about other internal and external factors, increase knowledge about which determinants of mental distress would be important to act on when particularly stressful conditions persist over time. If well-implemented, multifaceted strategies as well as specific interventions focused on the determinants of distress could contribute to improving the well-being of employees and society, which would inevitably result in an improvement in the quality of the assistance provided and, more generally, in the quality of life of the whole community.

## Figures and Tables

**Table 1:** Descriptive statistics of the study participants (*n* = 673).

		Physicians (*n* = 265)	Nurses (*n* = 176)	Other Healthcare Professionals (*n* = 184)	Administrative Staff (*n* = 48)	*p*
Sociodemographic variables						
Age		43.57 ± 10.96	45.96 ± 12.74	42.65 ± 11.79	47.28 ± 10.71	0.011
Sex	Men	44 (16.6%)	81 (46.0%)	52 (28.3%)	18 (37.5%)	<0.001
	Women	221 (83.4%)	95 (54.0%)	132 (71.7%)	30 (62.5%)	
Marital status	Single	35 (13.3%)	26 (14.8%)	40 (21.9%)	4 (8.3%)	0.214
	In a relationship	63 (23.9%)	50 (28.4%)	46 (25.1%)	11 (22.9%)	
	Married	131 (49.6%)	87 (49.4%)	84 (45.9%)	26 (54.2%)	
	Divorced or Separated	31 (11.7%)	11 (6.3%)	11 (6.01%)	6 (12.5%)	
	Widower	4 (1.4%)	2 (1.1%)	2 (1.09%)	1 (2.08%)	
PHQ-4 (Symptoms of Depression)		2 (0; 2)	2 (0; 2)	1 (0; 2)	1 (0; 2)	0.701
PHQ-4 (Symptoms of Anxiety)		2 (1; 3)	2 (1; 2)	2 (1; 2)	1 (1; 2)	0.141
IES-R		23 (13; 35)	17 (8.5; 27.5)	18 (9; 31.5)	21 (12; 33)	0.002
MBI-GS		45.15 ± 12.34	43.63 ± 11.59	43.09 ± 13.49	44.52 ± 13.50	0.118

Notes. Age and MBI-GS are presented as mean ± standard deviation; sex and marital status are presented as frequency and percentages (in round brackets); PHQ-4 (both symptoms of depression and anxiety) and IES-R are presented as median and interquartile ranges (in round brackets).

**Table 2:** Symptoms of depression cross-validation results.

	Physicians		Nurses		Other Healthcare Professionals		Administrative Staff	
	% Selected	% Confirmed	% Selected	% Confirmed	% Selected	% Confirmed	% Selected	% Confirmed
Perceived stress during the last two weeks	100	100	63	76	97	100	83	91
Job satisfaction during the last two weeks	95	96	77	94	-	-	-	-

Notes. % Selected: % of times independent predictors were selected in the training set. % Confirmed: % of times independent predictors were tested for significance in the testing set. “-” means that the possible predictor was not selected and confirmed in at least 70% of the time.

**Table 3:** Symptoms of anxiety cross-validation results.

	Physicians		Nurses		Other Healthcare Professionals		Administrative Staff	
	% Selected	% Confirmed	% Selected	% Confirmed	% Selected	% Confirmed	% Selected	% Confirmed
Perceived stress during the last two weeks	100	100	97	100	99	100	97	92
Job satisfaction during the last two weeks	93	95	-	-	-	-	-	-

Notes. % Selected: % of times independent predictors were selected in the training set. % Confirmed: % of times independent predictors were tested for significance in the testing set. “-” means that the possible predictor was not selected and confirmed in at least 70% of the time.

**Table 4:** Post-traumatic stress symptoms cross-validation results.

	Physicians		Nurses		Other Healthcare Professionals		Administrative Staff	
	% Selected	% Confirmed	% Selected	% Confirmed	% Selected	% Confirmed	% Selected	% Confirmed
Perceived stress during the last two weeks	77	96	89	99	85	98	-	-
Pyschological support since COVID-19 beginning of pandemic	-	-	86	93	-	-	-	-
Lack of recreative activities after work	-	-	-	-	-	-	73	78

Notes. % Selected: % of times independent predictors were selected in the training set. % Confirmed: % of times independent predictors were tested for significance in the testing set. “-” means that the possible predictor was not selected and confirmed in at least 70% of the time.

**Table 5:** Burnout symptoms cross-validation results.

	Physicians		Nurses		Other Healthcare Professionals		Administrative Staff	
	% Selected	% Confirmed	% Selected	% Confirmed	% Selected	% Confirmed	% Selected	% Confirmed
Perceived stress during the last two weeks	98	99	82	95	-	-	-	-
Impact of COVID-19 on daily work	82	93	-	-	-	-	-	-

Notes. % Selected: % of times independent predictors were selected in the training set. % Confirmed: % of times independent predictors were tested for significance in the testing set. “-” means that the possible predictor was not selected and confirmed in at least 70% of the time.

## Data Availability

The database is available at: https://doi.org/10.5281/zenodo.5818476 (accessed on 4 January 2022).

## Supplementary Materials

The following supporting information can be downloaded at: https://www.mdpi.com/article/10.3390/ijerph192013712/s1, The Supplementary materials contain: the stepwise logistic regression results (Tables S1–S5). Table S1: Psychological symptoms: cutoff frequencies; Table S2: Symptoms of depression (PHQ-2) stepwise regression analysis; Table S3: Symptoms of anxiety (GAD-2) stepwise regression analysis; Table S4: Post-traumatic stress symptoms (IES-R) stepwise regression analysis; Table S5: Burnout symptoms (MBI-GS) stepwise regression analysis. File S1: The COVID-19 Survey.
