# Supplementary material for: Prevalence of Psychopathological Symptoms and Their Determinants in Four Healthcare Workers’ Categories during the Second Year of COVID-19 Pandemic
Source: Int J Environ Res Public Health. 2022 Oct 21;19(20):13712. doi: 10.3390/ijerph192013712 (PMC9602535; doi:10.3390/ijerph192013712)
Supplement: Supplementary file 1 [file ijerph-19-13712-s001.zip › File S1.pdf]

File S1
The COVID-19 Survey

1. **Write your age in numbers below (es. 45)**

____

2. **Select your sex:**
- o Man
- o Women

3. **Select your current marital status:**
- o Single
- o In a relationship
- o Married
- o Separated or Divorced
- o Widowed

4. **Write your current employment:**

____

5. **Select your employment contract:**
- o Permanent contract
- o Fixed-term contract
- o Stand-in assignment
- o Volunteering
- o Other: ____________________________________

6. **Do you suffer from one or more organic disease/s? Select one or more options from the list below <u>if and only if</u> you have one or more medical diagnosis/es:**
- o No, I do not suffer from an organic disease
- o Yes, I suffer from respiratory system disease/s (e.g. asthma, chronic obstructive pulmonary disease, etc.)
- o Yes, I suffer from immune system disease/s (e.g. thyroiditis, psoriasis, rheumatoid arthritis, etc.)
- o Yes, I suffer from oncologic disease/s
- o Yes, I suffer from metabolic disease/s (e.g. diabetes, gout, obesity, etc.)
- o Yes, I suffer from liver disease/s (e.g. hepatitis, cirrhosis, etc.)
- o Yes, I suffer from kidney disease/s
- o Yes, I suffer from cardiovascular disease/s
- o Yes, I suffer from psychiatric disease/s
- o Yes, I suffer from other disease/s: ____

7. **Are you implementing any strategy to protect your family from COVID-19 infection?**
- o Yes, I live away from home or my family live away form home (i.e. hotel room)
- o Yes, I stay in a separated room in the same house
- o Yes, I use PPE at home
- o No, I cannot implement any strategy at home
- o No, I think there is no more reason to do this
- o Other: ____

8. **Have you ever been tested positive to COVID-19 swab?**
- o Yes, I am positive right now
- o Yes, I have, but now I am negative
- o No, I have not

9. **Have you got the COVID-19 vaccination?**
- o Yes, I have
- o No, I have not

10. **How scared do you feel about contracting COVID-19 after the vaccination? Move the bar on the line between 0 (No scared at all) to 100 (Extremely scared):**

0 10 20 30 40 50 60 70 80 90 100

______________________________|______________________________

11. **Have you ever thought about starting a psychological support since the COVID-19 pandemic beginning?**
- o No, I have never thought about that
- o Yes, I have, but I do not have started it yet
- o Yes, and I have started a psychological support

12. **Have you been working in shifts since the start of the pandemic?**
- o Yes
- o No

13. **Have you worked in a COVID-19 ward since the start of the pandemic?**
- o Yes, but only during the first wave (March-June 2020)
- o Yes, but only during the second wave (September-December 2020)
- o Yes, I am still working in a COVID-19 ward
- o No, I have never worked in a COVID-19 ward

14. **How much COVID-19 is still impacting your daily work activities?**
- o Not at all
- o A little bit
- o Moderately
- o Quite a bit
- o Extremely

15. **How much COVID-19 is still impacting your daily work activities? We provide you several reasons. For each one, move the bar on the line between 0 (Strongly disagree) to 100 (strongly agree)**

1	Bureaucracy increased	0 10 20 30 40 50 60 70 80 90 100
2	Extended working hours	0 10 20 30 40 50 60 70 80 90 100
3	More pressure or demands	0 10 20 30 40 50 60 70 80 90 100
4	Tense working mood	0 10 20 30 40 50 60 70 80 90 100
5	Lack of contact and support from both colleagues and superiors	0 10 20 30 40 50 60 70 80 90 100
6	Lack of recreative activities after work	0 10 20 30 40 50 60 70 80 90 100

7	Increased family burden	0 10 20 30 40 50 60 70 80 90 100
8	Clinical different opinions about patients' treatments	0 10 20 30 40 50 60 70 80 90 100
9	Prolonged PPE used	0 10 20 30 40 50 60 70 80 90 100
10	Routineous work	0 10 20 30 40 50 60 70 80 90 100
11	Lack of information and involvement in clinical decisions	0 10 20 30 40 50 60 70 80 90 100
12	Greater responsibilities and risks in dealing with COVID-19 patients	0 10 20 30 40 50 60 70 80 90 100
13	Lack of psychological and emotional support from extra-working context (i.e. family, friends, significant other, etc.)	0 10 20 30 40 50 60 70 80 90 100

16. **Looking to the future, how much confidence do you feel towards…**

		**Not at all**	**A little bit**	**Moderately**	**Quite a bit**	**Extremely**
1	…the Italian healthcare system					
2	…your job perspectives					
3	…your perspective other than job (i.e. family, friends, etc.)					
4	…the Italian socioeconomic situation					

17. **I feel…by patients**

- o Loved
- o Appreciated
- o Ignored
- o Rejected
- o Assaulted
- o Other:____________________________

18. **I feel…by patients' families**

- o Loved
- o Appreciated
- o Ignored
- o Rejected
- o Assaulted
- o Other:______________________________

19. **I feel…by my family**

- o Loved
- o Appreciated
- o Ignored
- o Rejected
- o Assaulted
- o Other:______________________________

20. **I feel…by my friends**

- o Loved
- o Appreciated
- o Ignored
- o Rejected
- o Assaulted
- o Other:______________________________

21. **I feel…by my neighbors**

- o Loved
- o Appreciated
- o Ignored
- o Rejected
- o Assaulted
- o Other:______________________________

22. **I feel…by the public opinion**

- Loved
- Appreciated
- Ignored
- Rejected
- Assaulted
- Other:____________________________

23. **How much does the judgments of…affects you?**

		**Not at all**	**A little bit**	**Moderately**	**Quite a bit**	**Extremely**
1	The patients					
2	The patients' families					
3	Your family					
4	Your friends					
5	Your neighbors					
6	The public opinion					
