# Supplementary material for: Prevalence of Psychopathological Symptoms and Their Determinants in Four Healthcare Workers’ Categories during the Second Year of COVID-19 Pandemic
Source: Int J Environ Res Public Health. 2022 Oct 21;19(20):13712. doi: 10.3390/ijerph192013712 (PMC9602535; doi:10.3390/ijerph192013712)
Supplement: Supplementary file 1 [file ijerph-19-13712-s001.zip › Tables S1-S5.pdf]

Supplementary Materials

Table S1. Psychological symptoms: cutoff frequencies

		Physicians (n = 265)	Nurses (n = 176)	Other healthcare professionals (n = 184)	Administrative staff (n = 48)	p
**PHQ-4 (Symptoms of Depression)**	Absent	223 (40,33%)	139 (25,14%)	154 (27,85%)	37 (6,69%)	0,415
	Present	41 (34,75%)	37 (31,36%)	30 (25,42%)	10 (8,47%)	
**PHQ-4 (Symptoms of Anxiety)**	Absent	192 (37,65%)	138 (27,06%)	141 (27,65%)	39 (7,65%)	0,333
	Present	72 (44,72%)	38 (23,6%)	43 (26,71%)	8 (4,97%)	
**IES-R**	Absent	141 (35.07%)	118 (29.35%)	115 (28.61%)	28 (6.97%)	0.181
	Mild	49 (44.95%)	29 (26.61%)	24 (22.02%)	7 (6.42%)	
	Moderate	14 (41.18%)	8 (23.53%)	9 (26.47%)	3 (8.82%)	
	Severe	60 (47.62%)	21 (16.67%)	36 (28.57%)	9 (7.14%)	

Table S2. Symptoms of depression (PHQ-2) stepwise regression analysis

Physicians	**β**	**SE**	**p-value**
Perceived stress during the last two weeks	0,024	0,003	<.0001
Job satisfaction during the last two weeks	-0,015	0,003	<.0001
Psychological support since COVID-19 pandemic beginning (yes)	0,285	0,116	0.0246

Nurses	**β**	**SE**	**p-value**
Perceived stress during the last two weeks	0,013	0,004	0.0003
Job satisfaction during the last two weeks	-0,016	0,004	0.0003
Routineously working	0,007	0,003	0.0100
Lack of psychological and emotional support from both colleagues and superiors	0,012	0,003	0.0110
Impact of patients judgement on work	0,220	0,075	0.0471

Other Healthcare Professionals	**β**	**SE**	**p-value**
Perceived stress during the last two weeks	0,01<br>8	0,003	<.0001
Job satisfaction during the last two weeks	-0,013	0,004	0.0007
Lack of psychological and emotional support from both colleagues and superiors	0,007	0,003	0.0011
Trust in self-perspective other than work (i.e. family, friends, etc.)	-0,275	0,102	0.0485
Impact of public opinion judgement on work	0,242	0,077	0.0132

Administrative staff	**β**	**SE**	**p-value**
Perceived stress during the last two weeks	0,025	0,004	<.0001
Psychological support since COVID-19 pandemic beginning (yes)	1,283	0,390	0.0015
Impact of public opinion judgement on work	0,441	0,124	0.0009
Marital status	0,332	0,123	0.0084
Work contract	-1,011	0,321	0.0033

Table S3. Symptoms of anxiety (GAD-2) stepwise regression analysis

Physicians	**β**	**SE**	**p-value**
Perceived stress during the last two weeks	0,028	0,003	<.0001
Job satisfaction during the last two weeks	-0,015	0,003	<.0001

Nurses	**β**	**SE**	**p-value**
Perceived stress during the last two weeks	0,022	0,003	<.0001
Job satisfaction during the last two weeks	-0,014	0,004	0.0004
Impact of public opinion judgement on work	0,202	0,073	0.0040
Lack of psychological and emotional support from both colleagues and superiors	0,009	0,003	0.0362

Other Healthcare Professionals	**β**	**SE**	**p-value**
Perceived stress during the last two weeks	0,023	0,003	<.0001
Trust in Italian healthcare system	-0,339	0,086	0.0488
Impact of public opinion judgement on work	0,277	0,077	0.0266

Administrative staff	**β**	**SE**	**p-value**
Perceived stress during the last two weeks	0,035	0,005	<.0001
Age	0,036	0,012	0.0472
Lack of recreative activities after work	0,013	0,006	0.0519
Prolonged DPI use	-0,016	0,006	0.0155

Table S4. Post-traumatic stress symptoms (IES-R) stepwise regression analysis

Physicians	β	SE	p-value
Perceived stress during the last two weeks	0,183	0,040	<.0001
Fear of contracting COVID-19 after the vaccination	0,123	0,036	0.0001
Psychological support since COVID-19 pandemic beginning (yes)	6,261	1,480	0.0002
Age	0,229	0,085	0.0053

Nurses	β	SE	p-value
Perceived stress during the last two weeks	0,160	0,034	<.0001
Psychological support since COVID-19 pandemic beginning (yes)	7,315	1,468	0.0003
Trust in Italian socioeconomic situation	-3,100	0,861	0.0060
Lack of psychological and emotional support from both colleagues and superiors	0,118	0,029	0.0452
Impact of neighbours judgement on work	2,396	0,773	0.0069

Other Healthcare Professionals	β	SE	p-value
Perceived stress during the last two weeks	0,147	0,039	<.0001
Job satisfaction during the last two weeks	-0,156	0,041	0.0020
Lack of psychological and emotional support from both colleagues and superiors	0,120	0,031	0.0236
Fear of contracting COVID-19 after the vaccination	0,090	0,039	0.0033
Impact of patients judgement on work	3,259	0,753	<.0001

Administrative staff	β	SE	p-value
More pressure or demands	0,187	0,044	0.0413
Lack of recreative activities after work	0,301	0,046	0.0002
Lack of information and involvement in making decisions	0,103	0,052	0.0083
Trust in Italian job perspectives	-3,374	1,058	0.0723

Table S5. Burnout symptoms (MBI-GS) stepwise regression analysis

Physicians	β	SE	p-value
Perceived stress during the last two weeks	0,146	0,028	<.0001
Lack of information and involvement in making decisions	0,061	0,021	0.0122
Psychological support since COVID-19 pandemic beginning (yes)	2,691	1,021	0.0180
Impact of COVID-19 on working (yes)	2,635	0,666	<.0001

Nurses	β	SE	p-value
Perceived stress during the last two weeks	0,140	0,030	0.0046
Fear of contracting COVID-19 after the vaccination	-0,089	0,032	0.0118
Lack of information and involvement in making decisions	0,102	0,026	<.0001
Clinical different opinions about patients' treatments	0,070	0,027	0.0062

Other Healthcare Professionals	β	SE	p-value
Perceived stress during the last two weeks	0,154	0,034	<.0001
Job satisfaction during the last two weeks	-0,138	0,035	<.0001
Lack of contact and support from both colleagues and superiors	0,125	0,031	<.0001
Routineous work	-0,074	0,032	0.0179

Administrative staff	β	SE	p-value
Perceived stress during the last two weeks	0,234	0,047	<.0001
Trust in Italian job perspectives	-5,212	1,342	0.0010
Impact of public opinion judgement on work	3,313	1,562	0.0432
